# Development of the “Recovery from Eating Disorders for Life” Food Guide (REAL Food Guide) - a food pyramid for adults with an eating disorder

**DOI:** 10.1186/s40337-018-0192-4

**Published:** 2018-04-01

**Authors:** Susan Hart, Claire Marnane, Caitlin McMaster, Angela Thomas

**Affiliations:** 1 Nutrition Services, St Vincent’s Health Network, Darlinghurst, 2010 Australia; 2 The Boden Institute of Obesity, Nutrition, Exercise and Eating Disorders, University of Sydney, Camperdown, 2006 Australia; 3 Newtown Nutrition, Suite 1, 33 King St, Newtown, 2042 Australia; 4 Nutrition and Dietetics Program, The University of Sydney, Camperdown, 2006 Australia; 5 Weight Management Service, The Children’s Hospital at Westmead, Westmead, 2145 Australia; 6 Central Coast Eating Disorders Outpatient Service, Toukley, 2263 Australia

## Abstract

**Background:**

There is limited evidence to inform nutrition and dietetic interventions for individuals with eating disorders even though it is recommended as an essential part of multidisciplinary management. There is minimal guidance, an absence of standardised nutrition educational material, and no research on how best to educate patients on healthy eating and how to achieve nutrition adequacy. Therefore the REAL Food Guide was developed.

**Methods:**

The REAL Food Guide is a pyramid with four layers and key nutrition messages beside each layer that was conceived to address gaps in nutrition education and intervention for individuals with eating disorders. Written and verbal consumer feedback was obtained from consumers receiving treatment regarding the acceptability and usefulness of the REAL Food Guide. A unique database was developed to reflect the types of foods and realistic portion sizes that patients are likely to select. This database was used for nutrition modelling to assess the nutrition adequacy of three meal patterns (meat containing, vegetarian and semi-vegan) for both weight maintenance and weight regain. Each meal pattern was compared to the Nutrient Reference Values for Australia and New Zealand.

**Results:**

Nutritional analysis demonstrated nutritional adequacy of meal patterns for energy, macronutrients and most micronutrients when the recommended number of serves from the REAL Food Guide were assessed. All meal patterns were adequate in micronutrients except for the semi-vegan meal pattern that was inadequate in vitamin D. Feedback from individuals with eating disorders demonstrates the nutrition education tool was acceptable to them as they felt it was more helpful for their recovery than general nutrition guidelines.

**Conclusion:**

The REAL Food Guide is a comprehensive and user-friendly guide that clinicians can use to educate patients about components of a balanced and healthy diet. The guide can educate all eating disorder clinicians, including those who are new to the field, about the basics of nutrition. Clinicians using the guide can be confident that, if followed, patient’s energy and nutritional requirements will be met and important nutrition education messages are reinforced, that are tailored to the beliefs and concerns of individuals with eating disorders.

**Electronic supplementary material:**

The online version of this article (10.1186/s40337-018-0192-4) contains supplementary material, which is available to authorized users.

## Background

Despite eating disorders being a common mental health problem [1], accompanied by adverse physical and psychosocial health effects [2, 3], impaired quality of life [4], and a high risk of suicide [5], little research has evaluated nutrition and dietetic treatment options [6]. Eating disorders comprise individuals diagnosed with anorexia nervosa (AN), bulimia nervosa (BN), binge eating disorder (BED), and other specified feeding and eating disorders (OSFED) [7]. Consumers with AN typically, although not always, have a body mass index (BMI) below 17.5 kg/m^2^ achieved by rigid rules about food and severe dietary restriction, sometimes engaging in compensatory behaviours such as purging or excessive exercise [7]. Consumers with BN alternate between periods of restriction, followed by binge episodes where large amounts of food are consumed in an uncontrolled manner [7]. In Australia, the point prevalence of eating disorder behaviours has increased in the general population over a 10-year period [1]. Disruptions in eating patterns, diet quality and energy balance play a central role in the aetiology of eating disorders [8] with avoidance of adequate calorie intake as a core disturbance of the illness [9]. Examples of eating disorder behaviours include: energy restriction for weight loss [8, 10–14]; avoidance of specific foods particularly foods high in energy, perceived to be “fattening” or highly palatable [9, 15–18]; eating a limited range of foods [14, 17, 19]; restricting intake of dietary fat [8, 13, 20]; reduced flexibility with food [21]; filling up on low energy foods [16]; excessive use of artificial sweeteners [22–24]; excessive quantities of fruit and vegetables [24]; use of non-nutritive beverages such as water and diet soft drinks to suppress appetite and reduce the urge to binge eat [8, 13, 25]; and excessive intake of caffeine [8, 25–29]. Other disordered eating behaviours include: abnormal timing of meals and snacks [18]; over-involvement in food preparation, collecting recipes and menus [18]; excessive use of condiments [18]; overestimating meal portion sizes and confusion about how much to eat [14, 30].

Dietetic treatment aims to achieve a safe rate of weight restoration, reinstate or learn “normalised” eating behaviour, and provide nutrition education on the maintenance of long-term healthy eating essential for recovery [6, 18, 31–33]. It has been recommended by experts in the field that there should be greater emphasis on selecting a wide variety of foods including intake of energy-dense foods [19, 24], which are crucial to preventing relapse and must be continually encouraged and included throughout the course of treatment [19]. Other recommendations include: the inclusion of “feared” foods to slowly reduce anxiety associated with such foods [9, 31, 34, 35]; increased dietary intake of fats and oils daily [24]; a high intake of calcium-rich foods [32, 36] to improve bone density and prevent the early onset of osteoporosis [37]; and minimise intake of low energy and “diet” foods [32, 38–40]. Other interventions are aimed at increased flexibility of food choice [39], eating out socially [34] and eating for enjoyment rather than for weight control [38, 39, 41].

Evidence based guidelines on managing the nutritional needs of eating disorder patients, and details of how to implement recommendations into day-to-day dietetic practice are currently lacking [6, 17, 19, 42]. Some authors have stated that new approaches to treatment are clearly needed [9] as insufficient attention has been paid to addressing the optimal nutritional approaches in treatment [43], and inappropriate nutritional management may play a role in the protracted course experienced by some individuals [44]. There are descriptions of dietetic clinical practices available but no integration of this information into a meaningful intervention to establish a baseline of standardised practice [6, 42]. As a result dietetic practice and interventions for eating disorder patients are highly variable [36, 42, 45]. A Delphi study of 45 experienced nutrition providers with an average of 21 years working with eating disorder patients demonstrated only modest consensus on nutritional counseling practices in the treatment of AN [45]. There are few resources for dietitians that discuss weight restoration, meal planning and cognitive aspects of the disorder while remaining within scope of practice [45]. It is necessary to have a stronger evidence base to provide better and more consistent care [45]. In the absence of tailored education material, nutrition education guides designed for the whole community are usually adapted for education in the treatment of eating disorders. The Australian Guide to Healthy Eating (AGHE) [46] published in 2003 and updated in 2013, is the community level food selection guide in use in Australia. It is designed for the prevention of an overweight population, for reducing the risk of diet-related conditions (such as high blood pressure) and the risk of chronic diseases (such as Type 2 diabetes), reflecting prevalent health issues in Western populations [46]. Internationally, there are similar guides such as MyPlate in the USA [47] and the Eatwell Guide in the United Kingdom [48].

Using guides that are not tailored to the thinking patterns, beliefs and behaviours of the target group can make communication difficult [49]. Nutrition education programs and resources are more effective when they address motivation and behaviours affecting food choices of the target audience, rather than simply providing basic information [50, 51]. Tailoring the nutrition message means including relevant information, using familiar language, and taking into account values and beliefs of the target audience [50–52]. Tailored nutrition education guides have been developed for vegetarians [53], athletes [54], the aged [55], individuals with chronic medical conditions [56–58], and for specific cultural groups [59].

The REAL Food Guide (originally named the “Eating Disorders Healthy Eating Guide”) was developed by the first author (S.Hart) to help educate consumers who were seen for dietetic assessment and consultation. Focus groups were conducted (by C.Marnane) as a research project for a Master of Nutrition and Dietetics in 2011. The other authors (C.McMaster and A.Thomas) used the guide extensively with consumers between 2010 and 2017, refining and modifying its content, as well as disseminating its information via workshops and conferences.

This paper documents the development of the REAL Food Guide, a tailored nutrition education guide for eating disorder consumers, and evaluation of the nutrition messages conveyed by the REAL Food Guide compared to messages conveyed by a nutrition guide designed for the general population, the Australian Guide to Healthy Eating (2003 version; AGHE-03). The authors will also demonstrate that nutritional requirements can be met for most individuals with eating disorders requiring weight maintenance and weight regain, by following the number of serves and standardised portion sizes for each food group recommended by the REAL Food Guide. The target group for use with the REAL Food Guide is adult consumers with eating disorders of mild to moderate severity, being treated in the community or a partial hospitalisation setting.

## Methods

### Development of the REAL Food Guide

**Fig. 1:**
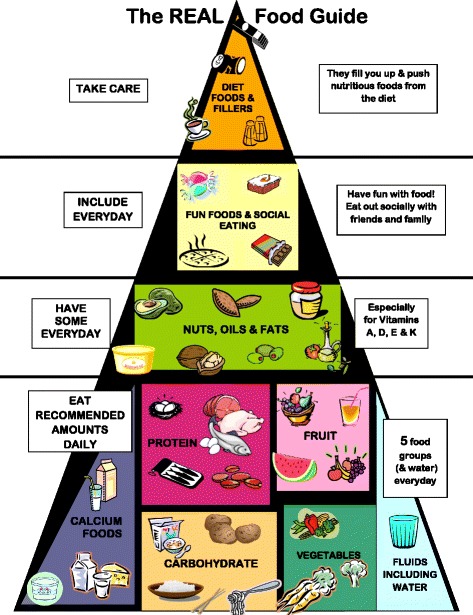
The REAL Food Guide

A review of nutrition and dietetic practice was undertaken with findings published previously [6, 25, 36, 42, 60]. An additional search of databases was undertaken for papers on nutrition intervention and recommendations for eating disorder patients published from 2011 to 2017. This literature, alongside the clinical experience of the authors was used to develop the REAL Food Guide (Fig. 1). All the dietetic authors have clinical experience in treating eating disorders across the service spectrum including specialist inpatient, partial hospitalisation (day program) and the outpatient setting, in both public and private facilities. Condensing this literature and collective clinical experience resulted in the REAL Food Guide.

### Evaluation of the REAL Food Guide

All adult consumers attending a specialist public hospital in Sydney, Australia for treatment of an eating disorder between August 2011 and October 2011 were invited to participate in focus groups evaluating the usefulness of the REAL Food Guide. Participants (*n* = 20) signed a consent form approved by the Human Research Ethics Committee of Sydney Local Health District for participation in focus groups and completion of a questionnaire of nutrition beliefs.

The sample was entirely female, and English speaking, with a mean age of 26.6 years (*SD* = 5.4), mean weight 59.7 kg (kg) (range = 38–103 kg) and mean Body Mass Index (BMI) of 23.7 kg/m^2^ (range = 14.5–37.7 kg/m^2^). Twelve participants were being treated for an eating disorder in an outpatient setting and eight in a day patient setting, which they attended daily for four days each week. Ten participants viewed the REAL Food Guide first and 10 viewed the AGHE-03 first. Thirteen consumers participated in one of two group sessions, with the remainder participating in individual sessions.

Oral presentations of two nutrition guides (AGHE-03 and REAL Food Guide) were delivered by one dietitian consisting of an explanation of each food group described in each nutrition guide, and the key nutrients those food groups provide. Specific information on portion sizes and number of daily serves for each food was not provided at this point in the evaluation phase. Guided discussion sessions were used to obtain participants’ opinions of the content of the nutrition guides with prompting questions such as: *What do you think the key nutrition message communicated by the guide was? What did you like and dislike about the guide? Is there anything you would change about the guide? Which guide would be more useful in working through an eating disorder?* A second researcher kept a written record of the verbal content of the discussion. The presentations were delivered in either group or individual format depending on participant availability. In the group format, participants observed a presentation of both the AGHE-03 and the REAL Food Guide, one week apart, with the order of presentations alternating with each successive group. For those viewing the guides individually, a presentation of one guide was given, and then the process was repeated with the second guide, with the research completed in a single session.

After each oral presentation of a food guide, participants completed a questionnaire assessing how well the guide had conveyed particular messages around healthy eating. Nine statements were included in the questionnaire, designed to capture key messages for eating disorder recovery such as food inclusivity and avoidance of negative messages around food (e.g. *“Carbohydrate is part of a healthy diet”*). Participants used a five-point Likert scale (strongly disagree, disagree, neutral, agree, strongly disagree) to rate how strongly each food guide conveyed each message. Due to the qualitative nature of the survey, and the relatively small number of participants, results have been presented without statistical analysis, allowing data to be assessed at face value.

### Nutritional adequacy of the REAL Food Guide

A database was developed for this study that combined nutrition composition data from several widely available databases [61–63]. A new and unique database was necessary to provide information required to meet study specifications, in order to reflect the types of food eating disorder consumers are more likely to select, as well as more realistic portion sizes than was found in existing databases. For example, clinical experience informs us that consumers would be more likely to choose “oatmeal” as opposed to “toasted muesli”, or “grilled salmon” as opposed to “roast beef”. Portion sizes in the new database were expressed as metric measures (i.e. 100 g), household measures (e.g. 1 cup, 2 teaspoons), or standard units (i.e. 2 slices of bread). Additionally, some portions were converted to hand measurements where the hand is used as a ruler, as described by Gibson, et al. [64]. If there was no data available for the portion size of a particular food, then all four authors weighed and measured the food portion. This was particularly important for using the hand as a ruler, where no lists of portion size were available.

Using this database, seven days of sample meal plans were constructed for consumers requiring weight regain, and those requiring weight maintenance. Three distinct meal patterns were analysed – meat containing, lacto-ovo vegetarian and vegan patterns. In total, six meal plans were developed and analysed, though only samples of five meal plans are presented in this paper (see the Additional files 1 and 2).

The meat containing meal pattern included red meat three times per week and fish three times per week. Dairy foods were the dominant calcium source used in the analysis. Additionally, a variety of unsaturated fats (nuts, margarine, olive oil, avocado) were included to align with health recommendations on increasing unsaturated fat in preference to saturated fat [65]. Other features of this meal pattern included hot meals in the evening, sandwiches for lunch five days per week, and two takeaway lunch meals. A total of six 250 ml cups of fluid (water, tea or coffee) were included in each sample meal plan. The lacto-ovo vegetarian meal pattern did not include meat, chicken or fish, rather alternative protein sources such eggs and cheese, with dairy foods as the dominant calcium source. The vegan meal pattern analysed did not include any animal proteins, and calcium-enriched soy products replaced dairy foods as the dominant calcium source. In reality, the vegan meal pattern is best described as “semi-vegan” as snack foods were included that were not specified as vegan. This semi-vegan pattern reflects limitations of hospital food services systems, where it is not practical or feasible to provide entirely vegan meals.

Adequacy of these meal plans was compared to the Nutrient Reference Values (NRVs) for Australia and New Zealand [65] for women aged 18 to 50 years, apart from calcium which used a higher target of 1300 mg (women 51 years and older) based on our awareness that there is earlier onset and a higher prevalence of osteoporosis in people with eating disorders [2, 37].

## Results

### Development of the REAL Food Guide

The bottom layer of the REAL Food Guide depicts five core food groups (fruit, vegetables, carbohydrate, protein, calcium foods), and fluid. The aim of the bottom layer of the REAL Food Guide is to encourage consumers to eat the same quantity of fruit and vegetables as represented in the community level guides, initially the AGHE-03 and then the AGHE-13. That is: two serves of fruits and five serves of vegetables daily, but not in excess of these amounts, as excessive intake is commonly observed in eating disordered individuals [24]. Dairy and calcium-enriched soy foods are depicted in the core foods section with the label of “Calcium Foods”. A key design element of the REAL Food Guide is the absence of a specific message on choosing reduced fat products, as seen in community nutrition guides such as the AGHE, MyPlate (USA) and the Eatwell Guide (UK) [46–48].

For protein, the guide aims to encourage inclusion of a variety of animal and vegetarian protein foods. Legumes are featured as an alternate protein source, and not featured in the vegetable and carbohydrate group (as with the AGHE) [46]. Our clinical experience informs us that our clients include legumes as a protein (for both vegetarians and non-vegetarians) but rarely as a carbohydrate, and almost never as a vegetable serve. The serve of legumes such as lentils in the REAL Food Guide is equivalent in energy (kilojoules) to meat, chicken, fish and eggs. Water is included with the core food groups to emphasise that adequate hydration is an important component of daily nutritional requirements, as fluid intake is often manipulated by eating disorder consumers as a weight control method [6].

The second layer of the REAL Food Guide describes “Nuts, Oils and Fats” to communicate that a healthy balanced diet includes adequate amounts of dietary fats and oils [66], and foods that contain them each day. Foods from this layer provide essential nutrients such as alpha linoleic acid, manganese and selenium***.*** Low dietary intake of vitamins A, D and E [12, 67–70] and deficiencies of essential fatty acids have been documented in consumers with eating disorders [20, 69, 70]. A written statement beside this layer indicates that this food group is necessary to provide fat-soluble vitamins A, D, E and K. On a practical level, it is difficult for individuals with AN to achieve an appropriate energy intake for weight regain when omitting foods containing fats and oils. Individuals with AN also need adequate dietary fat intake to return their body composition to normal levels for the return of menstruation [71].

The third layer of the pyramid is for foods consumed when eating out, during celebrations and as snacks, labelled as “Fun Foods and Social Eating”. These foods are included to assist with meeting dietary energy requirements and to challenge consumers’ beliefs about the inclusion of these foods, as they commonly believe that total abstinence is necessary for good health. It is also clinically important from a dietary and psychological perspective to include higher energy foods. Steinglass et al. [9] recommends targeting eating related anxiety by exposure to feared eating situations, which engages the patient in experiencing rather than avoiding their food fears, and the opportunity to experience habituation of anxiety and the disconfirmation of the feared consequence. Poor socio-emotional functioning and social isolation, driven in part by the avoidance of eating with others is a universal feature of eating disorders and has been suggested to be both a causal and maintaining factor of AN [72]. Treasure et al. [72] suggests that in addition to eating, interventions should target the social environment to produce change. Consumers frequently avoid social occasions that are anxiety provoking, may avoid eating with their family, and may eat alone which results in further social isolation. The REAL Food Guide recommends, “*Fun Foods be included daily, and eat out socially with your friends and family*” to practice making anxiety provoking choices with food. Consumers are encouraged to eat in a manner similar to the people they are eating with, and put a time limit on choosing foods off a menu.

The top layer of the REAL Food Guide is labelled “Diet Foods and Fillers”, which depicts low energy foods including caffeinated beverages and diet soft drinks, which are commonly used by eating disorder consumers to reduce hunger and suppress appetite [6, 8, 16, 22–24]. The key message is that excessive quantities of these foods may contribute to feelings of overfullness, and lead to difficulty in achieving recommended amounts of core food groups. The inclusion of diet foods is also counterproductive for weight restoration in consumers with AN. The recommendation is not necessarily to eliminate these foods but to “be careful” in regards to how they might affect overall dietary intake.

### Evaluation of the REAL Food Guide

Twenty participants who were receiving treatment for an eating disorder at Royal Prince Alfred Hospital, Sydney, participated in the focus groups to evaluate the key messages of the two food guides. Table 1 summarises the outcomes of the guided discussions. Broadly, the overall messages that participants felt the REAL Food Guide conveyed were “all foods are OK”, and “eat a variety of foods”. The key messages from the AGHE-03 were: “eat a variety of foods”, “eat less fats and oils” and “eat lots of carbohydrate”. Almost all participants (*n* = 19, 95%) felt the REAL Food Guide would be the more helpful of the two in facilitating recovery from an eating disorder.

**Table 1:** Summary of themes emerging from guided discussion with 20 participants

Prompting question asked by researcher	AGHE-03 (n)	REAL Food Guide (n)
What do you think is the key nutrition message?	Eat a variety of foods [15] Eat lots of carbohydrate [14] Eat less fats, oils and extra foods [11]	Eat a variety of foods [16] All foods are ok including fun foods and fats/oils [14]
Which guide do you think would be more helpful for working through an eating disorder?	AGHE-03 [1]	REAL Food Guide [19]
Why do you prefer this guide?	Nil response	• Increased relevance of content e.g. “It relates to the eating disorder. Like with the stuff as the top of the pyramid....you have your own little triggers and things, and the pyramid actually knows this” • Some felt the AGHE endorsed eating disorder cognitions, e.g. *“The message from the pie (the AGHE)* versus *the pyramid is about the same, but I look at this* (the AGHE) *and I know I could easily manipulate that for my eating disorder.”*
Placement of high energy snack foods, and fats and oils	• Participants disliked the placement of the extra foods separately to the core foods as they felt this indicated “*bad foods”* [15], and “*don’t eat these foods”* [16]. • Some liked the placement [3], because it reinforced their disordered beliefs: *“I’m happy with the extra foods being separate…. You can exclude these and never have them again”.* • Placement of fats and oils in the extras section was disliked by half the sample [10] e.g. *“They should say that there are good fats that you should have”.*	• Participants felt a sense of reassurance at seeing “fun foods” included in the REAL Food Guide, e.g. *“It’s good because it shows you it can be normal to eat small amounts of these foods every day, and that helps reduce any guilt you might feel about eating these foods”*.
Diet foods and fillers	N/A	Some participants liked the inclusion of diet foods and fillers [7] stating that *“this section is very relevant to people with eating disorders” and “you should think… why are you eating these? And that’s helpful”.*

The questionnaire evaluating how well each food guide conveyed certain messages around healthy eating are shown in Table 2. Compared to the AGHE-03, the REAL Food Guide was endorsed by more participants as supporting inclusivity of a broad range of foods, including fats/oils, “junk” or “fun” foods, whole dairy foods, red meat, and carbohydrates. Participants preferred the placement of fun foods in the REAL Food Guide compared with the placement of extras (called discretionary foods in AGHE-13), which were placed outside the image in AGHE-03*.* The inclusion of diet foods and fillers, which is unique to the REAL Food Guide, was also considered favourably*.* Participants perceived that both guides conveyed a similar message about fruit and vegetables, and fluid, specifically, “*eating a moderate amount of fruit and vegetables is part of a healthy diet*” and “*too much or too little fluid is not healthy*”.

**Table 2:** Participants who “agreed” or “strongly agreed” to messages conveyed by the AGHE-03 or the REAL Food Guide

Investigating nutrition messages communicated by each nutrition guide	Participants (*n*) who “agreed” or “strongly agreed” that this message was endorsed by AGHE-03	Participants (*n*) who “agreed” or “strongly agreed” that this message was endorsed by REAL Food Guide
Eat a variety of food every day	3	18
Junk food or fun foods can be included in a healthy diet	10	17
Whole dairy foods can be included in a healthy diet	3	18
Eating a moderate amount of fruit and vegetables is part of a healthy diet	10	8
Fats and oils can be included in a healthy diet	13	18
It’s not helpful to have diet foods to control my weight	7	14
Carbohydrate is part of a healthy diet	3	17
Red meat can be included in a healthy diet	4	19
Too much or too little fluid is not healthy	12	11

### Nutritional adequacy of the REAL Food Guide

Each of the six eating patterns fell within the Acceptable Macronutrient Distribution Ranges (AMDRs) for the macronutrients carbohydrate, protein and fat to reduce chronic disease risk whilst still ensuring adequate micronutrient status [65]. The AMDR for protein is to provide 15–25% of total energy intake daily, for dietary fat the AMDR is to provide 20–35% of total energy intake daily, and the AMDR for carbohydrate is to provide 45–65% of total energy intake daily. Additionally, all eating patterns met the NRVs for fibre, calcium, iron, zinc, sodium, and potassium [65]. An adequate fluid intake was achieved when six moderately sized cups of tea, coffee or water were included with each meal pattern [65]. Recommended number of serves of each core food group required to meet AMDRs and adequacy of micronutrients for weight regain and weight maintenance meal plans are listed in Table 3 (the detailed nutrition analysis for all meal patterns is in the Additional files 1 and 2). Both the weight maintenance and weight regain vegan eating patterns were not adequate in vitamin D but otherwise met all other NRVs [65]. The recommended serves per day for each core food group of the REAL Food Guide is listed in Table 3. These quantities are a guide and the authors recommend all individual nutritional requirements be assessed and reviewed by a Registered Dietitian as part of good clinical practice in the management of eating disorders [18, 31]. The recommended serving of each core food is an estimate of the minimum number of serves required each day for an adult older than 18 years, to achieve nutrient adequacy. It is a starting point, as some individuals on weight maintenance or weight regain may require more serves than is stated in this table. Increased requirements for energy result when individuals are physically active, are tall, male, have increased muscle mass, disproportionally more muscle mass than is expected for body weight, or other metabolic demands such as infection or illness. An adequate intake of fluid varies between individuals with increased fluid intake necessary to replace losses from vomiting or laxative use, from exercise, during hot weather, or with illness such as infection or fever.

**Table 3:** Recommended quantities of each food group for weight maintenance and regain

FOOD GROUP	Weight Maintenance Serves	Weight Regain Serves
CARBOHYDRATE meals (e.g. ½ cup cooked oatmeal, 2 slices bread, 1 cup cooked pasta)	3	4
CARBOHYDRATE snacks (e.g. 1 muesli bar, 1 slice bread)	1	2
PROTEIN (e.g. ½ cup grated cheese, ¾ cup beef mince, 1 cup tofu)	2	2
VEGETABLES/SALAD (e.g. ½ cup peas, 1 cup mixed salad)	4	4
FRUIT (e.g. 1 apple, 2 tablespoons raisins, 1 cup juice)	2	4
CALCIUM FOODS (e.g. 1 cup milk, 1 cup yoghurt)	3	4
NUTS, OILS & FATS (e.g. 1 teaspoon oil, 2 teaspoons margarine, 1 tablespoon avocado)	2	4
FUN FOODS (e.g. 3 chocolate coated biscuits, 3 scoops ice cream, 1/3 cup lollies)	1	1
FLUIDS (e.g. water, tea, coffee, juice)	1.5 to 2.0 Litres/day	1.5 to 2.0 Litres/day

## Discussion

The REAL Food Guide is a comprehensive, practical and user-friendly guide that eating disorder clinicians can use for patient education on consuming a balanced, healthy diet. A key strength of the guide is that it has been designed specifically for individuals with eating disorders, facilitating more targeted information on energy and nutrient needs, and also – crucially – addressing some common fears frequently observed in this population. Our research demonstrates that the REAL Food Guide is acceptable to individuals with eating disorders, with a clear preference for the REAL Food Guide as an aid to recovery being reported by this sample of 20 consumers in treatment for an eating disorder.

The REAL Food Guide may be used with several purposes in mind: for both consumer and clinician education; for nutritional assessment of consumers dietary intake; for benchmarking of food service delivery to programs that provide meals; and importantly, for research.

### Education

The REAL Food Guide is designed primarily as an education guide to illustrate components of a healthy diet and, uniquely, is tailored to the beliefs and attitudes that are endorsed by people with an eating disorder. The information is relevant to their concerns, so is more likely to be heard and understood by them, thereby facilitating improved communication [49, 52]. The qualitative evaluation of the REAL Food Guide (see Tables 1 and 2) showed a clear consumer preference for this guide in comparison to the AGHE-03 food guide, designed for use in the general population. All but one consumer nominated the REAL Food Guide, as the guide they thought would be better for their recovery. Key elements influencing outcomes involved the position or location of “fun foods”/“extra foods” and fats/oils in each of the nutrition guides. Consumers consistently felt the AGHE-03 conveyed the message that there are “*good” foods* and *“bad” foods,* primarily because of the placement and wording of the “extra” foods section. Rigidly dividing foods into “good” foods that may be eaten and “bad” foods that should be avoided, is a common eating disorder cognition [41, 44] manifesting in strict eating regimens. Challenging the good/bad food dichotomy with the message that all foods form part of a healthy diet is key to interventions that encourage more flexible eating.

It also emerged that the REAL Food Guide provided reassurance to participants by seeing an image that reinforced a rationale for eating high-energy foods and fats and oils. That is, the foods they had been restricting as a consequence of their eating disorder, but which they were now being encouraged to consume as part of their treatment and recovery.

From this research and from ongoing clinical use of the guide we are concerned that general community messages on nutrition that aim to minimise weight gain for the general population are inappropriate for nutrition education with eating disorder consumers, as they may reinforce eating disorder beliefs in this consumer group. Nutrition education guides, such as the AGHE-03 and AGHE-13, are designed for an entire population, primarily to help reduce the rate of overweight and obesity in the population [73]. We are concerned that this may result in harm to eating disorder consumers who internalise these messages and use them to justify their extreme dietary restriction.

As a result of the food modelling, the authors demonstrated that vegan meal plans for both weight maintenance and weight regain met the AMDRs and NRVs for Australian women (aged 18 to 50) except for vitamin D. The authors have included the weight maintenance vegan meal plan in the Additional files 1 and 2 but have not included the weight regain meal plan for publication (even though we analysed this plan for nutritional adequacy) as the restrictive nature of a vegan meal pattern could potentially interfere with a patient’s ability to gain weight. Currently there is a lack of research as to whether adhering to such a strict diet is helpful or harmful for consumers with an eating disorder, and it is unclear how this would impact on recovery from both a nutritional, and psychological point of view. Until the relationship between endorsement of a vegan eating pattern and recovery from an eating disorder is better understood, the authors currently do not recommend a vegan meal pattern for routine use in the treatment of eating disorders. We recommend that further research be undertaken regarding the feasibility of vegan meal patterns in the overall treatment and recovery process.

In addition to use with consumers, the REAL Food Guide could potentially be of use to dietitians new to the field of eating disorders, given the lack of training material available in this area. To date there has been no tailored resource that can be used to train and increase skills of non-dietitians’ knowledge of nutrition. This is a significant gap in the multidisciplinary management of eating disorders as Cordery and Waller [74] have stated that it is essential for every clinician working in the area to have sufficient basic knowledge of nutrition in order to effectively detect and challenge disordered thinking, beliefs and behaviour in their consumers. In their research they found that non-dietitians’ knowledge of nutrition was poor [74]. A standardised and simple education resource means that clinicians can have a consistent and evidence based message to refer to, particularly when they do not have access to a dietitian.

In regards to team dynamics and cohesion, when working with an eating disorder consumer it is essential for all involved clinicians to present a consistent, agreed upon and evidence based message. Building knowledge of basic nutrition requirements for treatment of eating disorders helps non-dietitians maintain accurate, consistent responses to consumer queries about meal plans and food [43, 74]. Whilst dietitians remain the expert in nutrition, all members of the treatment team will at times be pressed for answers about meal plans, and knowing how to respond to such queries is helpful [43, 74, 75]. In the absence of a consistent team message, non-dietitians may attempt to provide nutrition information that is inappropriate, inaccurate, or subject to personal beliefs about food [43, 74]. Inconsistent messages about food during treatment undermine trust in the treatment process [74]. It can result in more clinician time being devoted to discussions about minor food choices than other essential therapeutic interventions.

### Nutritional assessment

Nutritional analysis of the constructed standard (meat containing) and vegetarian meal plans demonstrates that nutritional adequacy can be achieved for weight maintenance and weight regain, and be used to compare individuals’ intakes against AMDRs and NRVs. This can be useful in a clinical setting (reviewing consumers’ intake using diet histories or food diaries) and for food service delivery (when auditing meals that are provided to consumers within intensive treatment settings). Clinicians using the guide can be confident that, *if followed,* the meal plans will meet consumer’s energy and nutritional requirements in the majority of cases and essential messages of nutrition education are reinforced. It is important to note that some consumers (e.g. those who are physically active, are above average height for age or are male) may require adjustments to their meal plan. Ideally changes to meal plans (as opposed to giving established nutrition messages) should always be done in collaboration or consultation with a dietitian who is an essential member of the multidisciplinary team in the management of eating disorders.

### Limitations

Limitations of the REAL Food Guide are that it has been evaluated and undergone nutrition modelling for adult women only, 18 years and older, with either AN, BN and OSFED. It has not been evaluated by people with BED, males, or those under 18 years. As Family Based Therapy is the first line treatment for children and adolescents with eating disorders it is unclear how the REAL Food Guide may work as an adjunct. It would be useful to evaluate the REAL Food Guide in the management of eating disorders in younger people and their families, however at this stage the authors do not recommend the REAL Food Guide be used for this age group. Additionally it is a limitation that all consumers in the study were from one hospital. Ideally, this study should be repeated using participants from multiple sites.

A further limitation is that the research with consumers was undertaken with the AGHE-03. In 2013 a revised edition of the Australian Dietary Guidelines (AGHE-13) was published. However, the AGHE-13 is similar to the AGHE-03 in regards to themes aimed at restriction, avoidance of fats/oils, “fun foods” (called “discretionary foods” in the AGHE-13), and minimising weight gain. As such, the negative comments made by participants in the focus groups remain relevant with the revised AGHE-13, although ideally this will be confirmed with further research.

Lastly, the REAL Food Guide was evaluated by participants attending outpatient or day program treatment, but not those in inpatient treatment. At the time of conducting the research, the patients on the inpatient unit were deemed too unwell to concentrate sufficiently, consequently the sample comprises participants who may be at a later “stage of change” in recovery. It is possible that those less ready for recovery may not view the REAL Food Guide as positively as those surveyed, however that does not preclude the contents of the guide from being appropriate for their health and nutritional needs.

## Conclusion

Evidence-based guidelines on managing the nutritional needs of eating disorder patients are lacking [6, 17, 19, 42], and new approaches to treatment are needed [9]. This research demonstrates that the tailored REAL Food Guide is more effective than a generalist food guide (the AGHE-03) at addressing common eating disorder nutrition messages, and is acceptable as a nutritional education guide to eating disorder consumers. This reproducible nutrition intervention that summarises all existing evidence to date, including recommendations [6, 12, 18], position statements [31] and expert consensus [45], will enable research into the effectiveness of nutrition and dietetic intervention to proceed. Previously, nutrition research in this area has used untested nutrition interventions, providing questionable outcome data regarding the effectiveness of nutrition intervention [76]. The anticipated results of future robust research will result in a stronger evidence base of effective and valid clinical interventions; the development of standardised nutrition and dietetic practices; and importantly, better and more consistent care for eating disorder patients [45].

## Additional files

Additional file 1: Portion Size. (DOCX 10488 kb)

Additional file 2: Nutrition analysis and food modelling. (DOCX 351 kb)
