# Supplementary material for: Development of the “Recovery from Eating Disorders for Life” Food Guide (REAL Food Guide) - a food pyramid for adults with an eating disorder
Source: J Eat Disord. 2018 Apr 1;6:6. doi: 10.1186/s40337-018-0192-4 (PMC5878939; doi:10.1186/s40337-018-0192-4)
Supplement: Supplementary file 1 — Portion Size. (DOCX 10488 kb) [file 40337_2018_192_MOESM1_ESM.docx]

Additional file 1 Portion Size


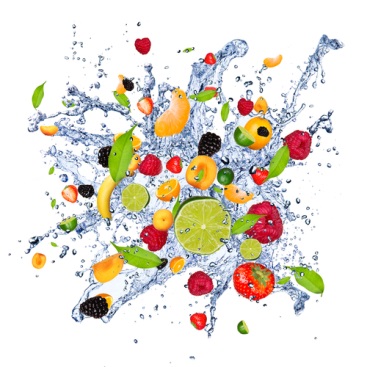


| **Recommended quantities of each food group for weight maintenance and regain ^1^** | | | | |
| --- | --- | --- | --- | --- |
| FOOD GROUP ^2^ | | Weight maintenance ^3^  Serves | | Weight regain ^3,4^  Serves |
| CARBOHYDRATE meals  *(e.g. ½ cup cooked oatmeal, 2 slices bread, 1 cup cooked pasta)* | | **3** | | **4** |
| CARBOHYDRATE snacks  *(e.g. 1 muesli bar, 1 slice bread)* | | **1** | | **2** |
| PROTEIN  *(e.g. ½ cup grated cheese, ¾ cup beef mince, 1 cup tofu)* | | **2** | | **2** |
| VEGETABLES/SALAD  *(e.g. ½ cup peas, 1 cup mixed salad)* | | **4** | | **4** |
| FRUIT  *(e.g. 1 apple, 2 tablespoons raisins, 1 cup juice)* | | **2** | | **4** |
| CALCIUM FOODS  *(e.g. 1 cup milk, 1 cup yoghurt)* | | **3** | | **4** |
| NUTS, OILS & FATS  *(e.g. 1 teaspoon oil, 2 teaspoons margarine, 1 tablespoon avocado)* | | **2** | | **4** |
| FUN FOODS  *(e.g. 3 chocolate coated biscuits, 3 scoops ice cream, 1/3 cup lollies)* | | **1** | | **1** |
| FLUIDS ^5^  *(e.g. water, tea, coffee, juice)* | | 1.5 to 2.0 Litres  /day | | 1.5 to 2.0 Litres /day |

**Notes on estimating the number of portions you, or a client needs each day**

1. It is ideal to review any dietary plan with a Dietitian. This guide is for general information and guidance only.
2. Energy intake as measured in kilocalories or kilojoules may vary considerably for individuals. Increased requirements for energy intake result when individuals are physically active. Other factors that increase energy requirements are: being tall, male, having increased muscle mass, disproportionally more muscle mass than is expected for your weight; if you are ill with infection or fever.
3. This is an estimate of the minimum number of serves required each day for an adult older than 18 years to achieve nutrient requirements. It is a starting point, and many individuals on weight maintenance or regain may require more serves than is stated in this table.
4. The authors define weight regain as being suitable for those individuals with a Body Mass Index less than 20 kg/m^2^.
5. The adequate intake of fluid varies considerably between individuals. Increased fluid intake is necessary to replace losses from vomiting or laxative use. Other factors that may increase fluid requirements are exercise, during hot weather, or illness such as infection or fever.
6. Unless otherwise stated all foods lists are for standard varieties and NOT fat modified, light, low fat or skimmed varieties.

| **CARBOHYDRATE (Meal portion)** | | |
| --- | --- | --- |
| *Carbohydrate refers to grains, cereal and some starchy vegetables such as potatoes. Choose a variety of whole grains and carbohydrate foods for dietary fibre, thiamine, folate and iodine. Carbohydrate is needed for your body to function, to stabilise your blood glucose level, and provide fuel for your muscles and brain. Your brain cannot use any other fuel to supply its energy demands other than glucose.* Eating less *carbohydrate than your body needs can lead to tiredness, fatigue, dizziness, irritability, and low blood glucose levels. It is recommended that at least 50% or more of the total energy of your diet should come from carbohydrate, the remaining energy provided by protein and essential fats and oils. The amount of carbohydrate you need is proportional to the amount of physical activity you do, and if you need to gain or maintain your weight.* | | |
| **Food** | **The size of 1 serve** | |
| Muesli or Granola | ½ cup | |
| Cereal flakes (i.e. Sustain) | 1 cup | |
| Bran cereal | 1 cup | |
| Oatmeal (cooked) | 1 cup | |
| Puffed cereal (i.e. Rice Bubbles) | 1 ½ cups | |
| Bread roll | 1 roll | |
| Bread roll, dinner size | 2 rolls | |
| Sliced bread | 2 slices | |
| Raisin toast | 2 slices | |
| Pasta (cooked) | 1 cup | |
| Spaghetti (raw) | Dry spaghetti strands as wide as a 10 cm coin | |
| Rice (cooked) | 1 cup | |
| Barley (cooked) | 1 cup | |
| Quinoa (cooked) | 1 cup | |
| Potato, baked or boiled | The size of your fist * | |
| Noodles thick, cooked | 1 ½ cups | |
| Sweet corn | 1 cup | |
|  |  |  |

**An example of one carbohydrate serve = ½ cup of muesli**


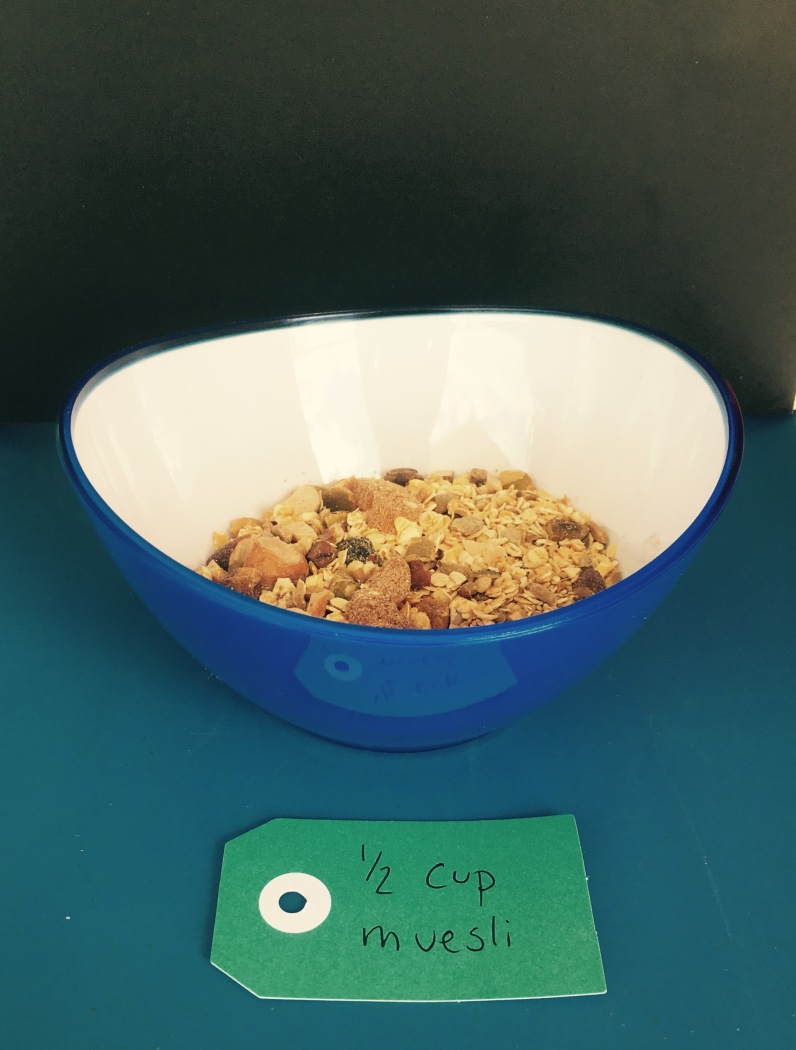


|  | **CARBOHYDRATE**  **(Snack portion)** |  |
| --- | --- | --- |

| **Food** | **The size of 1 serve** |
| --- | --- |
| Muesli bar | 1 bar |
| Popcorn | 4 cups |
| Crumpet + 1 teaspoon spread | 1 round |
| Bread + 1 teaspoon spread | 1 slice |
| Raisin toast + 1 teaspoon spread | 1 slice |
| Rice crackers | 16 crackers |
| Plain dried crackers (no spread) | 6 crackers |
| Plain dried crackers + 1 teaspoon spread | 4 small or 3 large crackers |

|  | **PROTEIN FOODS** | |  | |
| --- | --- | --- | --- | --- |
| *Protein rich foods provide iron, zinc, vitamin B12 and omega-3 essential fatty acids. Protein is needed for growth and repair of body tissues, and plays an important role in all functions of your body. If you are vegetarian, it is important to replace animal proteins with iron-rich substitutes. Additionally, iron found in non-meat foods is non-haem iron, which is poorly absorbed by the body. Ensuring you include a source of vitamin C with non-meat foods will help iron absorption.*   \| **Food** \| **The size of 1 serve** \| \| --- \| --- \| \| Sliced cheese \| 2 slices, the size of your palm * \| \| Grated cheese \| ½ cup \| \| Ricotta cheese \| ½ cup \| \| Chicken breast \| The size of your palm * \| \| Beef mince \| ¾ cup \| \| Fillet steak \| The size of your palm * \| \| Eggs \| 3 eggs \| \| Canned salmon, drained \| ½ cup \| \| Canned tuna, drained \| ¾ cup \| \| Fillet of fish, white \| The size of your whole hand* \| \| Tofu \| 1 cup \| \| Baked beans, tinned \| ¾ cup \| \| Chickpeas or kidney beans \| 1 cup \| \| Lentils, cooked \| 1 cup \| \| Almonds, whole \| 25, 1 full handful * \| \| Tahini \| 2 tablespoons \| \| Hummus \| 1/3 cup \| \| Peanut butter \| 2 tablespoons \| | | | | |
|  | |  | |  |

**An example of one serve of protein = one handful of almonds**

**
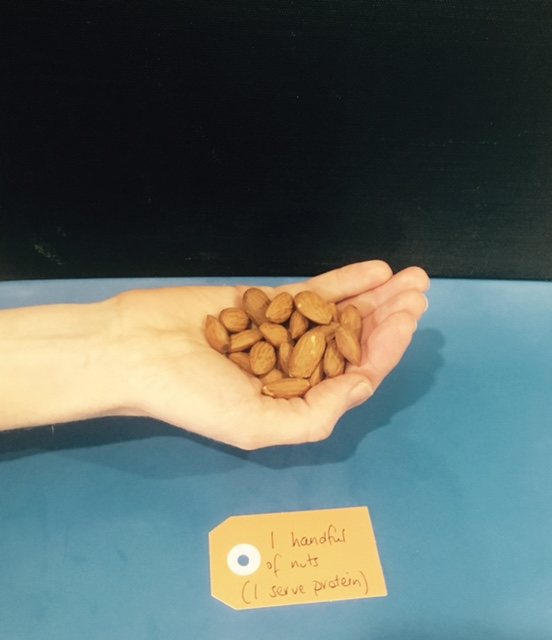
**

**An example of one protein serve = cheese the size of your palm the height or thickness of the cheese is one fingers width)**


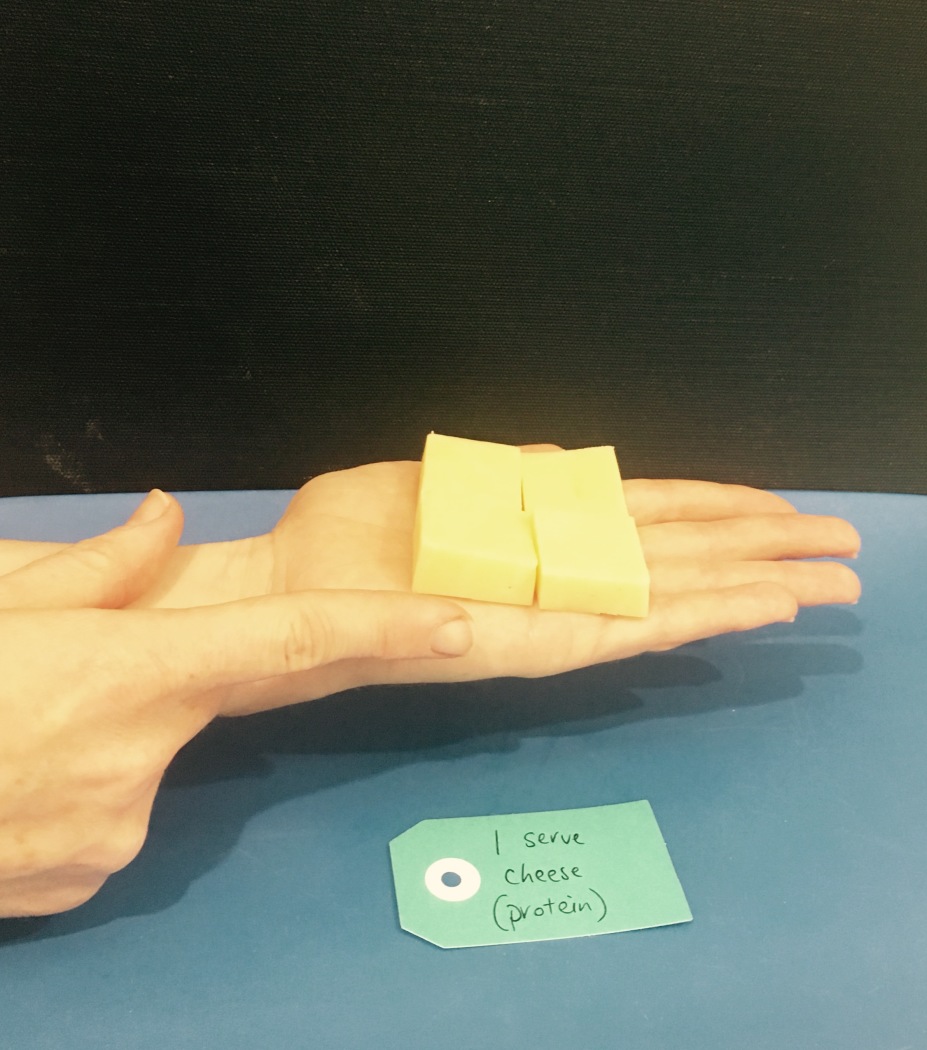


**An example of one serve of protein = chicken that fills 1/3 of your dinner plate**

**= a piece of chicken that fills the size of your palm**

**(note that this is the same piece of chicken)**


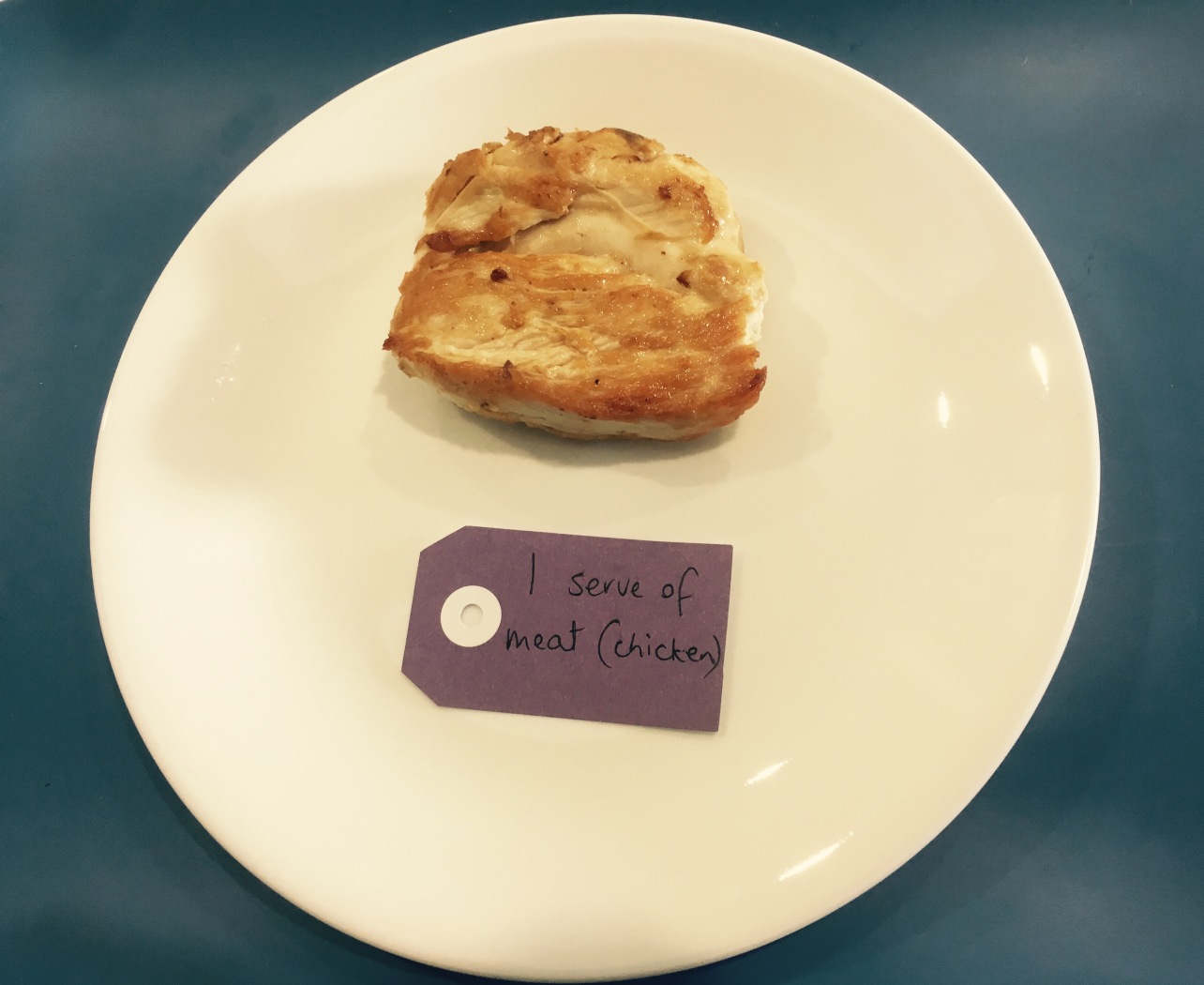


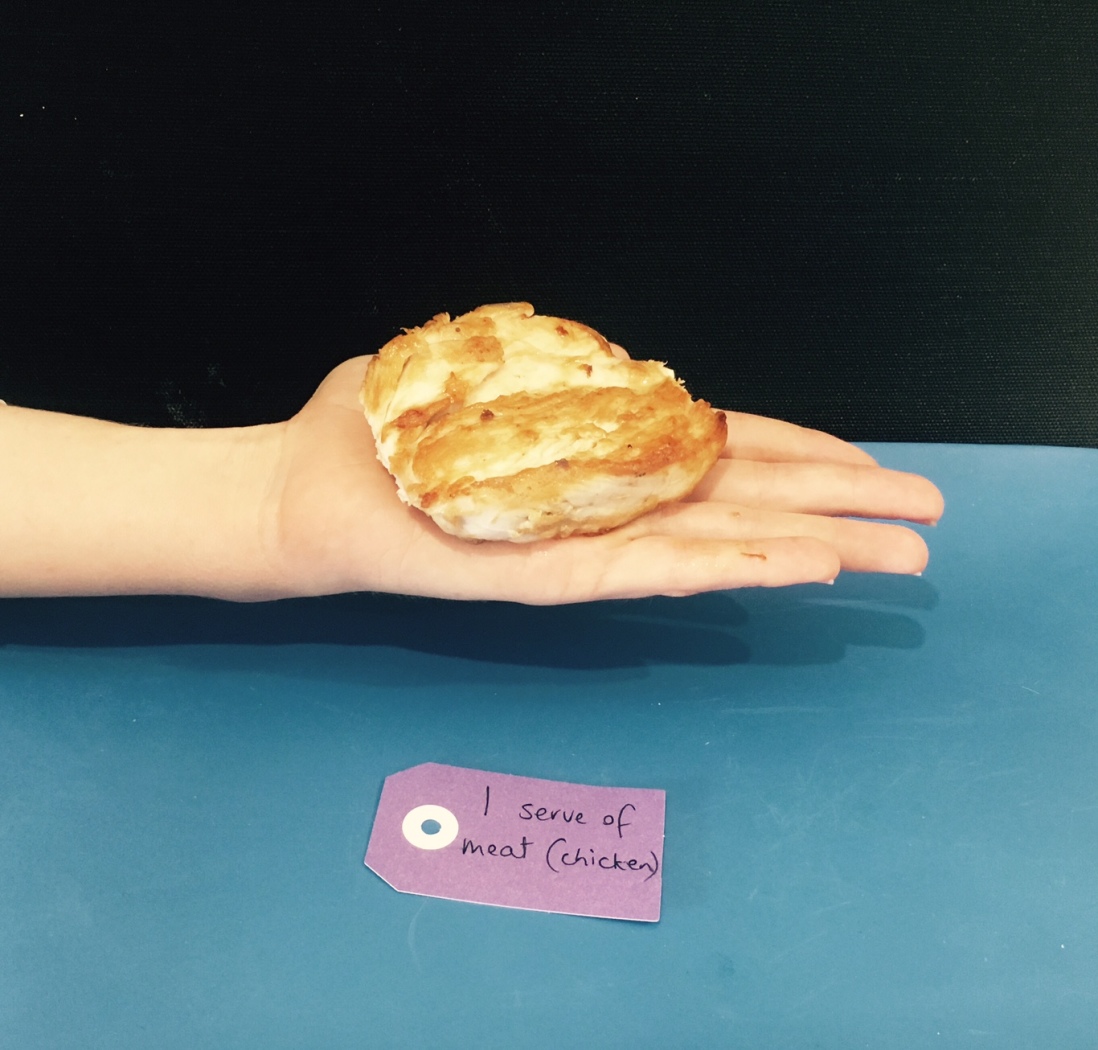


| **VEGETABLES/SALAD** | |
| --- | --- |
| *Choose a variety of vegetables with different colours to provide vitamin C, folate, potassium, beta-carotene and dietary fibre. It is important to not eat excessive quantities of these foods as they can fill you up, and push nutritious carbohydrate, fats and protein foods from your diet.* | |
| \| **Food** \| **The size of 1 serve** \| \| --- \| --- \| \| Mushrooms (raw) \| ½ cup \| \| Cucumber \| 1 piece the length of your index finger \| \| Capsicum (raw) \| ½ whole capsicum or the size of your fist \| \| Tomato \| 1 whole tomato the size of your fist  or 1 cup of cherry-sized tomatoes \| \| Mixed salad \| 1 cup \| \| Carrot (raw) \| 1 piece the length of your index finger \| \| Green peas (cooked) \| ½ cup \| |  |
| **FRUIT** |  |
| *Choose a variety of different fruits with different colours to provide potassium, dietary fibre vitamin C and other beneficial antioxidants. Try to include fruit in different forms, such as tinned, juiced and dried fruits, which are just as healthy as fresh fruit. It is important to ensure you don’t eat excessive quantities of fruit as it can fill you up, and push other foods from your diet affecting the overall nutrient balance of your diet. You can’t meet all your nutrition requirements from only a few food groups. One serve of fruit is the equivalent size to your fist.*   \| **Food** \| **The size of 1 serve** \| \| --- \| --- \| \| Orange, whole \| 1 \| \| Apple, whole \| 1 \| \| Pear, whole \| 1 \| \| Banana, small \| 1 \| \| Banana, large \| ½ \| \| Mango \| 1 \| \| Kiwifruit, whole \| 2 \| \| Mandarins \| 2 \| \| Plums \| 3 \| \| Grapes, bunch \| 3/4 cup \| \| Cherries \| 1 cup \| \| Raisins or sultanas \| 2 tablespoons \| \| Dried apricots \| 6 halves \| \| Tinned fruit, drained \| 1 cup \| \| Juice \| 1 cup \| \| Blueberries \| 1 cup \| \| Strawberries ** \| 2 cups \| | |

***Include strawberries only occasionally as 1 fruit serve, as the large portion can contribute to feeling full.*

|  | **CALCIUM FOODS** |  |
| --- | --- | --- |
| *Choose a variety of calcium containing foods for protein, riboflavin, and calcium. If you choose not to have dairy products ensure you choose an alternative that is fortified with calcium, as most do not naturally contain calcium. Also ensure the alternative has the equivalent energy value to dairy milk, otherwise you may be missing out and cutting some corners to achieving nutrient adequacy. Unless otherwise stated, all calcium foods refer to standard whole varieties and NOT modified versions such as light or skim milk. For example, 1 cup of whole plain milk is equivalent to over 2 cups of skim milk. As this is such a large serve, and you may also be missing out on some fat-soluble vitamins, we do not recommend these fat modified versions.*   \| **Food** \| **The size of 1 serve** \| \| --- \| --- \| \| Flavoured milk \| 1 cup \| \| Plain unflavoured milk \| 1 ¼ cups \| \| Soy milk, plain unflavoured \| 1 ½ cups \| \| Cheese \| 2 slices, the size of your palm * \| \| Grated cheese \| ½ cup \| \| Custard \| ¾ cup \| \| Flavoured yoghurt \| 1 cup \| \| Plain unflavoured yoghurt \| 1 cup \| | | |

|  | **NUTS, OILS and FATS** |
| --- | --- |
| *Foods containing essential fatty acids found, such as nuts, seeds, olives, unsaturated fats and oils (i.e. sunflower, olive and sesame oil) are essential for good health. It is recommended that one third or 30% of the total energy of the food you eat every day comes from dietary fat and oils. Foods that contain fat tend to be more tasty or “satiating” than low fat foods. Adequate amounts of dietary fat signal the brain that you have had enough to eat, which helps to give you the message to stop eating.*   \| **Food** \| **The size of 1 serve** \| \| --- \| --- \| \| Olive oil \| 1 teaspoon \| \| Margarine or butter \| 2 teaspoons \| \| Tahini (sesame seed spread) \| 2 teaspoons \| \| Peanut butter \| 2 teaspoons \| \| Cream cheese \| 3 teaspoons \| \| Avocado \| 1 Tablespoon \| \| Hummus \| 1 Tablespoon \| \| Olives \| 5 whole \| \| Nuts \| 6 almonds or 2 Tablespoons * \| | |

**An example of one serve of fats, oils and nuts = the size of 6 almonds**

(Note this is smaller than a protein serve shown above. For nuts smaller than almonds you will require greater than 6, and for nuts larger than almonds your will require less than 6)


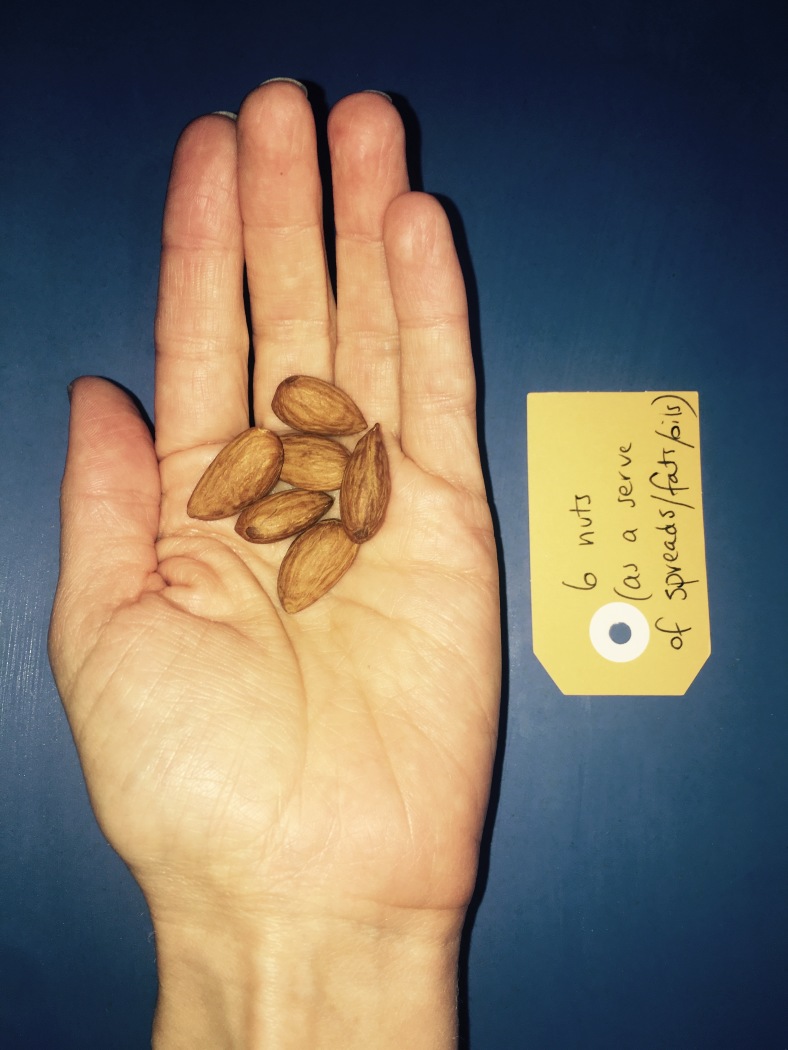


|  | **FUN FOODS** |  |
| --- | --- | --- |
| *Dietary guidelines recommend the inclusion of “fun foods or extras” in a balanced diet, particularly for people with higher energy requirements including adolescents, exercisers, and people needing to gain weight. Most people with eating disorders have lists of foods, which they refuse to allow themselves to eat. This can lead to rigid eating patterns, social isolation, and high levels of dietary restraint, all of which are unhelpful when trying to recover from an eating disorder. We recommend you challenge yourself to include these foods at least once each day, in order to expose yourself to the feelings and thoughts that emerge when you do so. This is a helpful step along your recovery journey.*   \| **Food** \| **The size of 1 serve** \| \| --- \| --- \| \| Rich chocolate biscuits \| 3 biscuits \| \| Cream biscuits \| 3 biscuits \| \| Wafer biscuits \| 4 biscuits \| \| Chocolate coated biscuits (i.e. Digestives) \| 4 biscuits \| \| Plain sweet biscuits \| 5 biscuits \| \| Chocolate coated honeycomb \| 5 pieces \| \| Chocolate buttons (i.e. M&Ms) \| 1 packet \| \| Chocolate bar \| 1 medium bar \| \| Kit Kat or wafer bar \| 1 bar (or 4 fingers of wafer) \| \| Lollies or sweets \| 1/3 cup \| \| Ice cream \| 3 scoops \| \| Crisps \| 1 single serve bag ~ 50 g \| \| Cupcake \| The bottom half fits neatly into ½ cup \| \| Muffin \| The bottom half fits neatly into ½ cup \| \| Piece of cake \| The size of your palm * \| | | |

**An example of one fun food = one medium sized cupcake or muffin**

(The size of the muffin or cupcake can fit the bottom half neatly into ½ cup (make sure you eat the whole cupcake with icing)

**
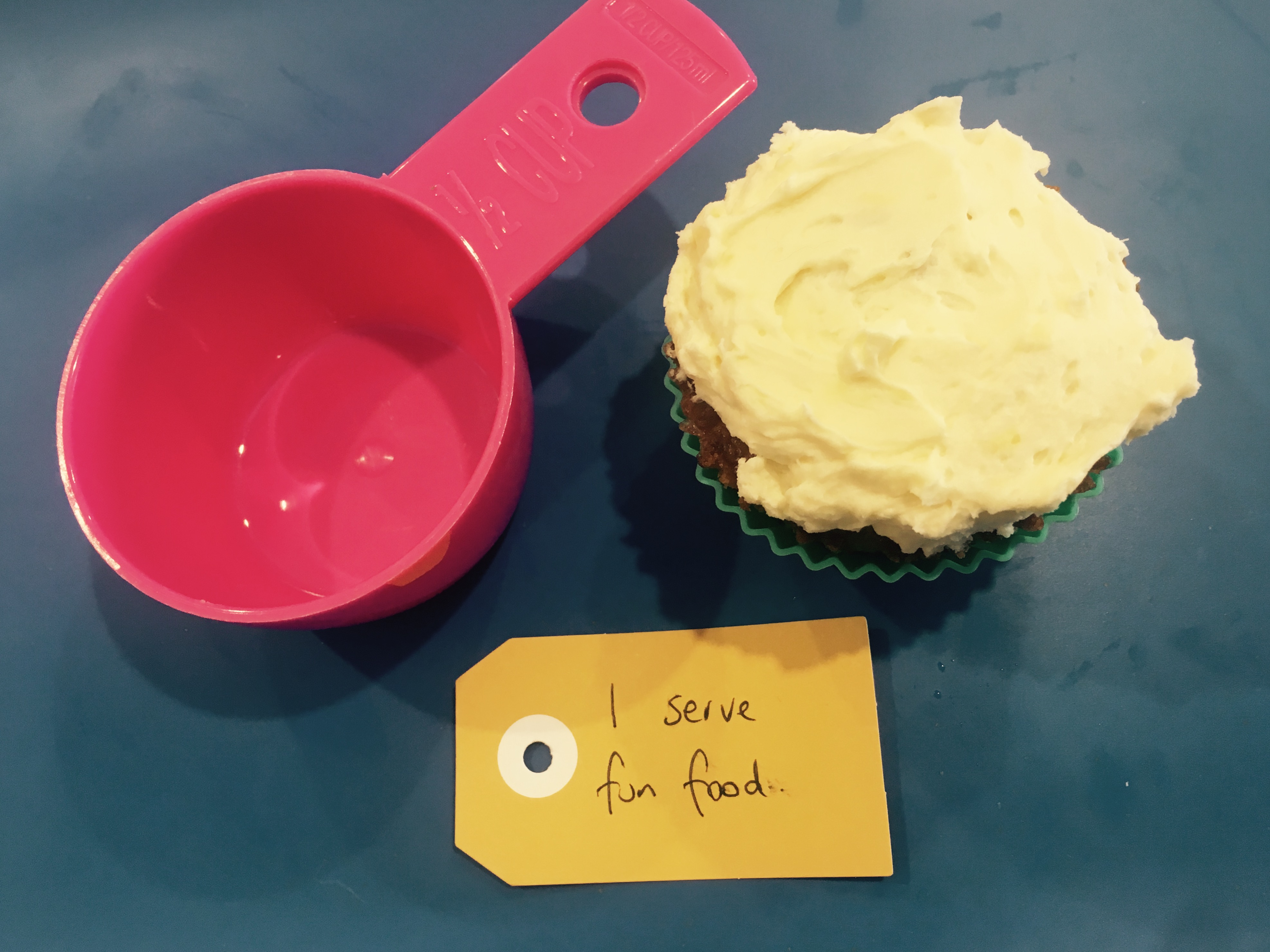
**

**An example of one fun food = 1/3 cup lollies / sweets**


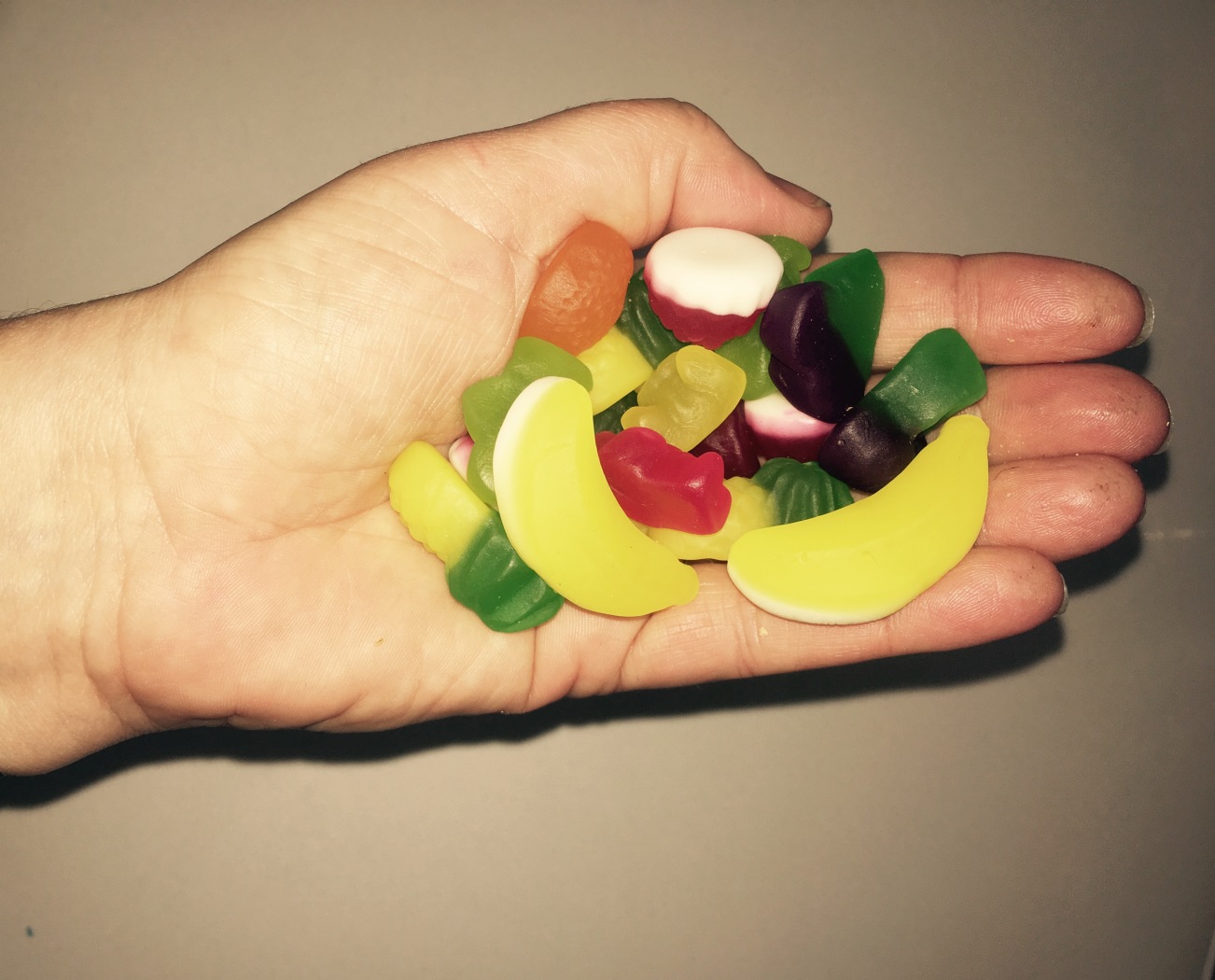


|  | **FLUIDS** |  |
| --- | --- | --- |
| *Pay attention to your fluid intake. Plan drinks into your day to ensure you don’t get too much or too little. As a rule of thumb, have one drink at each meal and snack throughout the day. More fluid is required if it is a hot day, you are exercising or you have increased fluid losses. Note, this group does not include alcohol.*  *The recommended serving size of “fluid” is 1 to 2 cups at each meal and snack, where 1 cup is 250 ml. This provides an adequate intake of fluid over the whole day.*   \| **Food** \| **Average serving serve** \| \| --- \| --- \| \| Glass of water \| 1 cup \| \| Glass of juice \| 1 cup \| \| Cup of coffee \| 1 cup \| \| Cup of tea \| 1 cup \| \| Can of soft drink \| 1 ½ cups \| | | |
|  |  | |
|  |  |  |

|  | **FILLERS & DIET FOODS**  **LIMIT OR NONE** |  |
| --- | --- | --- |
| *Sometimes people with eating disorders consume diet foods and fillers. From a nutrition standpoint there is nothing wrong with these foods; it’s just that when they make up a significant part of your daily intake, your nutrition needs are unlikely to be met. They often push out more nutritious foods from your diet, and may suppress your appetite. They may also keep you focussed on dietary rules and restricting food.*  *The following foods are example of diet foods and fillers:*   - Diet drinks - Diet cola - Foods labelled as “diet” - Artificial sweeteners - Artificially sweetened products - Chewing gum - Excessive servings of fruit, especially strawberries - Excessive servings of vegetables - Excessive quantities of sauces i.e. *Sweet chilli sauce, or tomato sauce* - Excessive quantities of salt/pepper - Fat-or energy modified foods - Foods with a “Health halo” i.e. products where a health benefit is attributed to the food - Sugar-free sweets. | | |

| *** PORTION ESTIMATION AIDS** |
| --- |

Estimating portion size is a skill that needs to be practiced and learnt. It isn’t something that should be left to chance, as the amount of food eaten may be under-estimated. More research needs to be done on the use of portions estimation aids in eating disorders. In the mean time, using visual aids such as photographs, or your hand (which can be used as a ruler) provides a reference point. The authors don't recommend you weigh or measure your food. Learning the skill of estimating portions is best done in conjunction with a Dietitian.

**Hand Measurements**

| **Part of hand** | **Description** |
| --- | --- |
| Palm | Length from the heal of palm to base of index finger |
| Index finger | Length from base to tip of index finger |
| Hand | Length from the heal of palm to just below your “pinky” or smallest finger |
| Fist | Make fist, medium tension (not lose not tight), measure longest point between base of thumb to 1st knuckle of pointer |
