# Supplementary material for: Development of the “Recovery from Eating Disorders for Life” Food Guide (REAL Food Guide) - a food pyramid for adults with an eating disorder
Source: J Eat Disord. 2018 Apr 1;6:6. doi: 10.1186/s40337-018-0192-4 (PMC5878939; doi:10.1186/s40337-018-0192-4)
Supplement: Supplementary file 2 — Nutrition analysis and food modelling. (DOCX 351 kb) [file 40337_2018_192_MOESM2_ESM.docx]

Additional file 2 NUTRITION ANALYSIS

& FOOD MODELLING

**
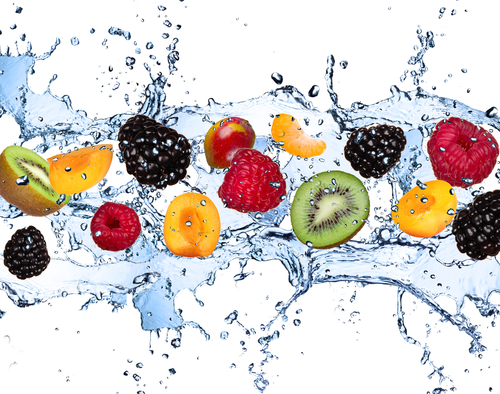
**

| **Recommended quantities of each food group for weight maintenance and regain ^1^** | | |
| --- | --- | --- |
| FOOD GROUP ^2^ | Weight maintenance ^3^  Serves | Weight regain ^3,4^  Serves |
| CARBOHYDRATE meals  *(e.g. ½ cup cooked oatmeal, 2 slices bread, 1 cup cooked pasta)* | **3** | **4** |
| CARBOHYDRATE snacks  *(e.g. 1 muesli bar, 1 slice bread)* | **1** | **2** |
| PROTEIN  *(e.g. ½ cup grated cheese, ¾ cup beef mince, 1 cup tofu)* | **2** | **2** |
| VEGETABLES/SALAD  *(e.g. ½ cup peas, 1 cup mixed salad)* | **4** | **4** |
| FRUIT  *(e.g. 1 apple, 2 tablespoons raisins, 1 cup juice)* | **2** | **4** |
| CALCIUM FOODS  *(e.g. 1 cup milk, 1 cup yoghurt)* | **3** | **4** |
| NUTS, OILS & FATS  *(e.g. 1 teaspoon oil, 2 teaspoons margarine, 1 tablespoon avocado)* | **2** | **4** |
| FUN FOODS  *(e.g. 3 chocolate coated biscuits, 3 scoops ice cream, 1/3 cup lollies)* | **1** | **1** |
| FLUIDS ^5^  *(e.g. water, tea, coffee, juice)* | 1.5 to 2.0 Litres /day | 1.5 to 2.0 Litres /day |

**Notes on estimating the number of portions you, or a client needs each day**

1. It is ideal to review any dietary plan with a Dietitian. This guide is for general information and guidance only.
2. Energy intake as measured in kilocalories or kilojoules may vary considerably for individuals. Increased energy requirements result when individuals are physically active. Other factors that increase energy requirements are: being tall, male, having increased muscle mass, or disproportionally more muscle mass than is expected for your weight, or if you are ill with infection or fever.
3. This is an estimate of the minimum number of serves required each day for an adult older than 18 years to achieve nutrient adequacy. It is a starting point, and many individuals on weight maintenance or regain may require more serves than is stated in this table.
4. The authors define weight regain as being suitable for those individuals with a Body Mass Index less than 20 kg/m^2^.
5. An adequate intake of fluid varies considerably between individuals. Increased fluid intake is necessary to replace losses from vomiting or laxative use. Other factors that may increase fluid requirements are exercise, during hot weather, or illness such as infection or fever.
6. Unless otherwise stated all foods lists are for standard varieties and NOT fat modified, light, low fat or skimmed varieties.

WARNING TO INDIVIDUALS WITH A LIVED EXPERIENCE

*The following document contains details on meal patterns suitable for treatment of people with an eating disorder.*

*We recommend this document to be used by or in collaboration with a clinician.*

*The document includes a detailed nutrient analysis demonstrating that each meal plan meets nutritional requirements. For some people with a lived experience of an eating disorder this detailed information may be triggering or unhelpful.*

*If you are interested in viewing this document we recommend you access this document with your dietitian and/or other treating health professionals.*

1a. STANDARD WEIGHT MAINTENANCE MEAL PATTERN

**Specifications of the meal pattern**:

- Main meals
  - Includes a variety of animal proteins and red meat three times per week at dinner
  - Fish meals are included three times per week
  - Hot main meals are included at dinner, once daily
  - Sandwiches are included at lunch five times per week
  - Two takeaway meals per week are included at lunch (chicken breast and chips; jacket potato and baked beans).
- Other
  - Includes dairy foods *(an alternative is calcium-enriched soy foods)*
  - Includes a variety of foods containing unsaturated fats (nuts, margarine, olive oil, hummus)
  - Includes six cups (250 mL) of fluid (water/tea/coffee) each day.

**1a. Meal plan showing serves of each core food group (maintenance)**

|  | **Recommended serves of core foods** | Sample food choices |
| --- | --- | --- |
| **BREAKFAST**  *Before 9 am* | **1 carbohydrate serve**  **1 calcium serve**  **1 fruit serve**  **1 fluid** | 1 bowl cereal  1 cup milk / tub yoghurt  Fresh fruit/fruit cup/juice  Tea / coffee |
| **AM SNACK** | **1 fruit serve**  **1 calcium serve**  **1 fluid** | 1 apple  1 tub yoghurt / 2 slices cheese  Tea / coffee / water |
| **LUNCH**  *Between 12 and 2 pm* | **1 carbohydrate serve**  **1 protein serve**  **1 fat/oil serve**  **1 vegetable serve**  **1 fluid** | 2 slices bread  1 chicken breast  1 tablespoon avocado  Grated carrot & lettuce  1 cup water |
| **PM SNACK** | **1 fun food**  **1 fluid** | 1 piece carrot cake  Tea / coffee / water |
| **DINNER**  *Between 6 and 8 pm* | **1 carbohydrate serve**  **1 protein serve**  **1 fat/oil serve**  **1 vegetable serve**  **1 fluid** | 1 cup cooked rice  3/4 cup beef mince  1 teaspoon sesame oil  Onion, capsicum & beans  1 cup water |
| **SUPPER** | **1 fruit serve**  **1 calcium serve**  **1 fluid** | 6 dried apricot halves / banana  1 cup hot chocolate  Tea / coffee / water |

**1a. Nutrient analysis of maintenance meal plan containing red meat**

|  | **AVERAGE DAY** | **NUTRITION TARGET** |
| --- | --- | --- |
| **Energy** | 2098 kcal (8770 kJ) | 2000 - 2200 kcal |
| **Protein** | 24% energy (102 g) | 20% energy |
| **Carbohydrate** | 48% energy (255 g) | 50% energy |
| **Total Fat** | 28% energy (66 g) | 30% energy |
| **Fibre** | 36 g | > 25 g |
| **Calcium** | 1415 mg | > 1300 mg |
| **Iron** | 14.9 mg | 8 mg (EAR) |
| **Zinc** | 11.9 mg | 8 mg |
| **Vitamin D** | 4.8 ug | 5 ug |
| **Sodium** | 1980 mg | < 2300 mg |
| **Potassium** | 4738 mg | > 2800 mg |
| **Water** | 3.1 L | 2.8 L  (Adequate intake) |

1b. STANDARD WEIGHT REGAIN MEAL PATTERN

**Specifications of meal pattern**

- Main meals
  - Includes a variety of animal proteins and red meat three times per week at dinner
  - Fish meals are included three times per week
  - Hot main meals are included at dinner, once daily
  - Sandwiches are included at lunch five times per week
  - Two takeaway meals per week are included at lunch (chicken breast and chips; jacket potato and baked beans)
  - Two cups of juice (250 mL) are included daily, one at lunch and one at dinner.
- Snacks
  - Nut bars (approximately 50 g) are included three times each week (note: this option is a larger serving size than one handful of nuts or fat/oil serve)
  - Supper snacks include a serving of nuts three times per week, and a serving of fruit four times per week.
- Other
  - Includes dairy foods *(an alternative is calcium-enriched soy foods)*
  - Includes a variety of foods containing unsaturated fats (nuts, margarine, olive oil, hummus)
  - Includes four cups (250 mL) of fluid (water/tea/coffee) each day (with two cups of water optional).

**1b.** **Meal plan showing serves of each core food group (weight regain)**

|  | **Recommended serves of core foods** | Sample food choices |
| --- | --- | --- |
| **BREAKFAST**  *Before 9 am* | **2 carbohydrate serves**  **1 fat/oil serve**  **1 calcium serve**  **1 fruit serve**  **1 fluid** | 1 bowl cereal  2 slices bread + spread  1 cup milk / tub yoghurt  Fresh fruit / fruit cup / juice  Tea / coffee |
| **AM SNACK** | **1 carbohydrate snack**  **1 calcium serve**  **1 fluid** | Muesli bar or 16 rice crackers  1 tub yoghurt / 2 slices cheese  Tea / coffee / water |
| **LUNCH**  *Between 12 and 2 pm* | **1 carbohydrate serve**  **1 protein serve**  **1 fat/oil serve**  **1 vegetable serve**  **2 fruit serve** | 2 slices bread  1 chicken breast  1 tablespoon avocado  Grated carrot & lettuce  1 orange  1 cup juice |
| **PM SNACK** | **1 fun food**  **1 calcium serve**  **1 fluid** | 1 piece carrot cake  1 glass flavoured milk  Tea / coffee / water |
| **DINNER**  *Between 6 and 8 pm* | **1 carbohydrate serve**  **1 protein serve**  **1 fat/oil serve**  **1 vegetable serve**  **1 fruit serve** | 1 cup cooked rice  3/4 cup beef mince  1 teaspoon sesame oil  Onion, capsicum & beans  1 cup juice |
| **SUPPER** | **1 fruit serve**  **1 calcium serve**  **1 fluid** | 6 dried apricot halves / banana  1 cup hot chocolate  Tea / coffee / water |

1b. **Nutrient analysis of weight regain meal pattern containing red meat**

|  | **AVERAGE DAY** | **NUTRITION TARGET** |
| --- | --- | --- |
| **Energy** | 2850 kcal (11,900 kJ) | 2800 kcal |
| **Protein** | 22% energy (126 g) | 20% energy |
| **Carbohydrate** | 50% energy (361 g) | 50% energy |
| **Total Fat** | 28% energy (88 g) | 30% energy |
| **Fibre** | 45 g | > 25 g |
| **Calcium** | 1810 mg | > 1300 mg |
| **Iron** | 18.1 mg | 8 mg (EAR) |
| **Zinc** | 14.3 mg | 8 mg |
| **Vitamin D** | 5.9 ug | 5.0 ug |
| **Sodium** | 2363 mg | < 2300 mg |
| **Potassium** | 6067 mg | > 2800 mg |
| **Water** | 3.2 L | 2.8 L  (Adequate intake) |

2a. VEGETARIAN WEIGHT MAINTENANCE MEAL PATTERN

**Specifications of meal pattern**

- Main meals
  - Includes animal proteins seven times each week (from eggs and cheese) and a variety of vegetarian proteins seven times week (lentils, beans, tofu, chickpeas) at lunch and dinner
  - Red meat, fish and chicken are excluded
  - Hot main meals are included at dinner, once daily
  - Sandwiches are included at lunch five times per week
  - Two takeaway meals per week at lunch (vegetarian burger and chips; jacket potato and baked beans).
- Other
  - Includes dairy foods *(an alternative is calcium-enriched soy foods)*
  - Includes a variety of foods containing unsaturated fats (nuts, margarine, olive oil, hummus)
  - Includes six cups (250 mL) of fluids (water/tea/coffee)
  - Education is required on iron bioavailability.

**2a. Meal plan showing serves of core food groups (vegetarian maintenance)**

|  | **Recommended serves of core foods** | Sample food choices |
| --- | --- | --- |
| **BREAKFAST**  *Before 9 am* | **1 carbohydrate serve**  **1 calcium serve**  **1 fruit serve**  **1 fluid** | 1 bowl cereal  1 cup milk / tub yoghurt  Fresh fruit / fruit cup / juice  Tea / coffee |
| **AM SNACK** | **1 fruit serve**  **1 calcium serve**  **1 fluid** | 1 small banana  1 tub yoghurt / 2 slices cheese  Tea / coffee / water |
| **LUNCH**  *Between 12 and 2 pm* | **1 carbohydrate serve**  **1 protein serve**  **1 fat/oil serve**  **1 vegetable serve**  **1 fluid** | 1 bread roll  3 eggs mashed  Mayonnaise  Lettuce  1 cup water |
| **PM SNACK** | **1 fun food**  **1 fluid** | 3 rich chocolate biscuits  Tea / coffee / water |
| **DINNER**  *Between 6 and 8 pm* | **1 carbohydrate serve**  **1 protein serve**  **1 fat/oil serve**  **1 vegetable serve**  **1 fluid** | 1 cup cooked rice  1 cup lentils / chickpeas  Avocado  Lettuce, tomato & carrot  1 cup water |
| **SUPPER** | **1 fruit serve**  **1 calcium serve**  **1 fluid** | 1 cup tinned fruit salad  1 bowl ice cream  Tea / coffee / water |

2a. **Nutrient analysis of vegetarian, weight maintenance meal pattern**

|  | **AVERAGE DAY** | **NUTRITION TARGET** |
| --- | --- | --- |
| **Energy** | 2094 kcal (8750 kj) | 2000 - 2200 kcal |
| **Protein** | 20% energy (85 g) | 20% energy |
| **Carbohydrate** | 51% energy (267 g) | 50% energy |
| **Total Fat** | 28% energy (66 g) | 30% energy |
| **Fibre** | 40 g | > 25 g |
| **Calcium** | 1545 mg | > 1300 mg |
| **Iron** | 15.0 mg | 8 mg (EAR) |
| **Zinc** | 10.1 mg | 8 mg |
| **Vitamin D** | 4.5 ug | 5 ug |
| **Sodium** | 1687 mg | < 2300 mg |
| **Potassium** | 4450 mg | > 2800 mg |
| **Water** | 3.1 L | 2.8 L  (Adequate intake) |

2b. VEGETARIAN WEIGHT REGAIN MEAL PATTERN

**Specifications of meal pattern**

- Main meals
  - Includes animal proteins seven times each week (from eggs and cheese) and a variety of vegetarian proteins seven times week (lentils, beans, tofu, chickpeas) at lunch and dinner
  - Red meat, fish and chicken are excluded
  - Hot main meals are included at dinner, once daily
  - Sandwiches are included at lunch five times per week
  - Two takeaway meals per week are included at lunch (vegetarian patty and chips; jacket potato and baked beans)
  - One cup of juice (250 mL) is included at lunch and dinner
  - Two cups of juice (250 mL) are included daily, one at lunch and one at dinner.
- Snacks
  - Nut bars (approximately 50 g) are included three times each week (note: this option of nut bar is a larger serving size than one handful of nuts or fat/oil serve)
  - Supper snacks include one serving of nuts three times per week, and one serving of fruit three times per week.
- Other
  - Includes dairy foods *(an alternative is calcium-enriched soy foods)*
  - Includes a variety of foods containing unsaturated fats (nuts, margarine, olive oil, hummus)
  - Includes four cups (250 mL) of fluid (water/tea/coffee) each day (with two optional cups of water)
  - Education is required on iron bioavailability.

**2b. Meal plan showing serves of core food groups (vegetarian weight regain)**

|  | **Recommended serves of core foods** | Sample food choices |
| --- | --- | --- |
| **BREAKFAST**  *Before 9 am* | **2 carbohydrate serves**  **1 fat/oil serve**  **1 calcium serve**  **1 fruit serve**  **1 fluid** | 1 bowl cereal  2 slices toast + spread  1 cup milk / tub yoghurt  Fresh fruit / fruit cup / juice  Tea / coffee |
| **AM SNACK** | **1 carbohydrate snack**  **1 calcium serve**  **1 fluid** | Raisin toast / popcorn  1 tub yoghurt / 2 slices cheese  Tea / coffee / water |
| **LUNCH**  *Between 12 and 2 pm* | **1 carbohydrate serve**  **1 protein serve**  **1 fat/oil serve**  **1 vegetable serve**  **2 fruit serve** | 1 bread roll  3 eggs mashed  Mayonnaise  Lettuce  1 apple  1 cup juice |
| **PM SNACK** | **1 fun food**  **1 calcium serve**  **1 fluid** | 3 rich chocolate biscuits  1 flavoured milk  Tea / coffee / water |
| **DINNER**  *Between 6 and 8 pm* | **1 carbohydrate serve**  **1 protein serve**  **1 fat/oil serve**  **1 vegetable serve**  **1 fruit** | 1 cup cooked rice  1 cup lentils / chickpeas  Avocado  Lettuce, tomatoes & carrot  1 cup juice |
| **SUPPER** | **1 fruit serve**  **1 calcium serve**  **1 fluid** | 1 cup tinned fruit salad  1 bowl ice cream  Tea / coffee / water |

2b. **Nutrient analysis of vegetarian, weight regain meal pattern**

|  | **AVERAGE DAY** | **NUTRITION TARGET** |
| --- | --- | --- |
| **Energy** | 2800 kcal (11,690 kj) | 2800 kcal |
| **Protein** | 19% energy (107 g) | 20% energy |
| **Carbohydrate** | 52% energy (363 g) | 50% energy |
| **Total Fat** | 29% energy (89 g) | 30% energy |
| **Fibre** | 50 g | > 25 g |
| **Calcium** | 1975 mg | > 1300 mg |
| **Iron** | 18.5 mg | 8 mg (EAR) |
| **Zinc** | 12.9 mg | 8 mg |
| **Vitamin D** | 6.0 ug | 5.0 ug |
| **Sodium** | 1926 mg | < 2300 mg |
| **Potassium** | 5780 mg | > 2800 mg |
| **Water** | 3.2 L | 2.8 L  (Adequate intake) |

3a. VEGAN WEIGHT MAINTENANCE MEAL PATTERN

**Specifications of the meal pattern**:

- Main meals
  - Animal proteins are excluded at all main meals
  - Includes a variety of vegetarian proteins seven times week (lentils, beans, tofu, chickpeas) at lunch and dinner
  - Includes meat substitutes three times per week
  - When using nuts or nut spread as a protein choice, the larger serving size is required for nutrient adequacy (i.e. the serving size of nuts listed as a protein equivalent rather than as a snack equivalent)
  - Hot main meals are included at dinner, once daily
  - Sandwiches are included at lunch five times per week
  - Two takeaway meals per week are included at lunch (vegetarian burger and chips; jacket potato and baked beans).
- Snacks
  - Snack foods used are standard varieties and are not necessarily vegan i.e. chocolate bars. However, dairy free varieties can be substituted in as long as they are an energy equivalent to a standard snack variety.
- Other
  - Includes calcium-enriched soy foods
  - Includes a variety of foods containing unsaturated fats (nuts, margarine, olive oil, hummus)
  - Includes six cups (250 mL) of fluid (water/tea/coffee) each day
  - No dietary vitamin D is provided by this meal pattern, so serum levels should be monitored. Supplementation of vitamin D may be required as advised by your doctor or dietitian. Some foods such as soy milk may be fortified with vitamin D which will increase dietary intake of vitamin D
  - Education is required on iron bioavailability.

**3a. Meal plan showing serves of core food groups (vegan maintenance)**

|  | **Recommended serves of core foods** | Sample food choices |
| --- | --- | --- |
| **BREAKFAST**  *Before 9 am* | **2 carbohydrate serves**  **1 calcium serve**  **1 fruit serve**  **1 fluid** | 1 bowl cereal  1 cup soy milk  Fresh fruit / fruit cup / juice  Tea / coffee |
| **AM SNACK** | **1 fruit serve**  **1 calcium serve**  **1 fluid** | 1 apple / banana  1 tub soy yoghurt  Tea / coffee / water |
| **LUNCH**  *Between 12 and 2 pm* | **1 carbohydrate serve**  **1 protein serve**  **1 fat/oil serve**  **1 vegetable serve**  **1 fluid** | 1 wrap  2 large or 3 small falafels  Hummus  Lettuce  1 cup water |
| **PM SNACK** | **1 fun food**  **1 fluid** | 1 chocolate bar  Tea / coffee / water |
| **DINNER**  *Between 6 and 8 pm* | **1 carbohydrate serve**  **1 protein serve**  **1 fat/oil serve**  **1 vegetable serve**  **1 fluid** | 1 cup cooked rice  1 cup tofu  1 teaspoon oil  Zucchini & capsicum  1 cup water |
| **SUPPER** | **1 fruit serve**  **1 calcium serve**  **1 fluid** | 1 banana  1 glass flavoured soy milk  Tea / coffee / water |

3a. **Nutrient analysis of vegan weight maintenance meal pattern**

|  | **AVERAGE DAY** | **NUTRITION TARGET** |
| --- | --- | --- |
| **Energy** | 2079 kcals (8690 kj) | 2000 – 2200 kcals |
| **Protein** | 21% energy (89 g) | 20% energy |
| **Carbohydrate** | 51% energy (270 g) | 50% energy |
| **Total Fat** | 28% energy (62 g) | 30% energy |
| **Fibre** | 48 g | > 25 g |
| **Calcium** | 1524 mg | > 1300 mg |
| **Iron** | 21.8 mg | 8 mg (EAR) |
| **Zinc** | 11.6 mg | 8 mg |
| **Vitamin D** | < 1.0 ug | 5 ug |
| **Sodium** | 1740 mg | < 2300 mg |
| **Potassium** | 5745 mg | > 2800 mg |
| **Water** | 3.2 L | 2.8 L  (Adequate intake) |

**ABBREVIATIONS**

cm centimetre

EAR Estimated Average Requirement

g grams

kcal kilocalories

kj kilojoule

L Litres

mg milligram

mL millilitre

NRVs Nutrient Reference Values

ug micrograms
